# Cost-Effectiveness of Sequential Teriparatide/Zoledronic Acid Compared With Zoledronic Acid Monotherapy for Postmenopausal Osteoporotic Women in China

**DOI:** 10.3389/fpubh.2022.794861

**Published:** 2022-02-24

**Authors:** Ruxu You, Jinyu Liu, Lei Ke, Guangyi Yu, Yu Zhang, Takahiro Mori

**Affiliations:** ^1^Department of Pharmacy, Union Hospital, Tongji Medical College, Huazhong University of Science and Technology, Wuhan, China; ^2^Department of Pharmacy, Tongji Hospital, Tongji Medical College, Huazhong University of Science and Technology, Wuhan, China; ^3^Department of Pharmacy, People's Hospital of Dongxihu District, Wuhan, China; ^4^Department of General Medical Science, Graduate School of Medicine, Chiba University, Chiba, Japan; ^5^Health Services Research and Development Center, University of Tsukuba, Tsukuba, Japan; ^6^Department of General Internal Medicine, Eastern Chiba Medical Center, Togane, Chiba, Japan

**Keywords:** sequential therapy, teriparatide, zoledronic acid, cost-effectiveness analysis, postmenopausal osteoporosis

## Abstract

**Objective:**

We aimed to assess the cost-effectiveness of sequential teriparatide/zoledronic acid relative to zoledronic acid monotherapy for postmenopausal osteoporotic women in China.

**Methods:**

A previously validated Markov microsimulation model was updated to examine the cost-effectiveness of daily subcutaneous teriparatide for 2 years followed by annual intravenous zoledronic acid for 3 years (sequential teriparatide/zoledronic acid), compared with zoledronic acid monotherapy for 3 years in Chinese women with postmenopausal osteoporosis at ages 65, 70, 75, and 80 from the health care payer perspective.

**Results:**

The incremental cost-effectiveness ratios (ICERs) (US dollars [$] per quality-adjusted life-year [QALY]) of sequential teriparatide/zoledronic acid vs. zoledronic acid monotherapy was $173,223/QALY at age 65 years, which was much higher than the pre-determined willingness-to-pay (WTP) threshold of $ 31,512/QALY, and the results were similar at other ages. In one-way sensitivity analyses, the two most impactful parameters were the cost of teriparatide and the residual effects of the medications included in this study. Sequential teriparatide/zoledronic acid became cost-effective at age 80 with the cost of teriparatide reduced by 50%. Without the residual effect, the ICER increased to $257,982/QALY. Probabilistic sensitivity analyses shown that the probabilities of zoledronic acid monotherapy being cost-effective were 100% at a WTP of $31,512/QALY.

**Conclusions:**

Among Chinese women with postmenopausal osteoporosis, sequential teriparatide/zoledronic acid was not cost-effective unless the cost of teriparatide was reduced by 50% only for the participants over 80 years.

## Introduction

Osteoporosis is characterized by bone micro-architectural deterioration of bone tissue, leading to loss of bone mass. It is associated with an increased risk of fragility fractures, causing significant morbidity, mortality, and a substantial economic burden for society worldwide (1, 2).

Remarkable progress has been made toward developing new treatment options for osteoporosis. Early studies focused on antiresorptive medications for bone resorption, including bisphosphonates, denosumab, and so on. Such drugs have shown significant anti-fracture benefits, but there are risks that restrained their long-term use (3, 4). Firstly, they inhibit bone resorption for coupling between bone resorption and formation, but subsequently reduce formation. Secondly, the use of bisphosphonates and denosumab are associated with rare adverse reactions including the jaw and atypical femoral fracture.

Similarly, numerous studies focused on anabolic agents that enhance the anabolic activity of bone cells. The drug firstly approved by the National Medical Products Administration in China was teriparatide, which is a parathormone analog that stimulates osteoblast activity and was indicated in postmenopausal women with fragility fractures (5). However, the risk of osteosarcoma with teriparatide observed in initial preclinical studies in rats restricts its use to 2 years (6). In addition, the study found that in the months and years after treatment with teriparatide, some or all of the bone gained during treatment appears to be lost if no other therapy is implemented (7).

Therefore, recent studies have begun to focus on sequential treatments by using medications with different mechanisms of action for the treatment of osteoporosis (7, 8). In most studies, sequential therapy with an antiresorptive agent (e.g., denosumab or bisphosphonate) after the completion of an anabolic agent (e.g., teriparatide) was tested with promising results (9, 10). As shown in the clinical trials with extension/post-marketing follow-up, bisphosphonate after the completion of teriparatide can prevent a decline in bone mineral density (BMD) and, is even associated with an increase in BMD in some cases (11). However, the choice of therapy should be based on effectiveness, safety, cost, convenience, and other patient-related factors comprehensively. The annual cost of teriparatide is much higher than other anti-osteoporotic drugs, and to our knowledge, whether the sequential treatment of teriparatide is economical has not been studied in China.

Consequently, we aimed to evaluate the pharmacoeconomic of sequential treatment with teriparatide followed by zoledronic acid compared with zoledronic acid monotherapy and with no treatment among postmenopausal women with osteoporosis in China.

## Methods

### Overview

A previously validated Markov microsimulation model (12, 13) was updated and adapted to evaluate the cost-effectiveness of teriparatide followed by zoledronic acid (i.e., teriparatide/zoledronic acid) compared to zoledronic acid monotherapy and to no treatment in Chinese postmenopausal women at four different ages of treatment initiation (i.e., 65, 70, 75, and 80). The total health care costs and quality-adjusted life-years (QALYs) over a lifetime horizon (i.e., until an individual died or reached the age of 105 years) in each group were assessed, and the incremental cost-effectiveness ratios (ICERs), net monetary benefit (NMB), and net health benefit (NHB) were obtained from the Chinese health care payer perspective. Annual discount rates of 3% were used for health outcomes and costs according to the Chinese guidelines (14). The willingness-to-pay (WTP) in this study was pre-determined at three times the gross domestic product (GDP) per capita value of China in 2020 ($31,512). In line with the recent recommendations for the conduct of economic evaluation in osteoporosis (15) and the Consolidated Health Economic Evaluation Reporting Standards (CHEERS) statement (16), TreeAge Pro 2019 (TreeAge Pro Inc., Williamston, MA, USA) was used for building our model.

### Model Structure

**Figure 1:**
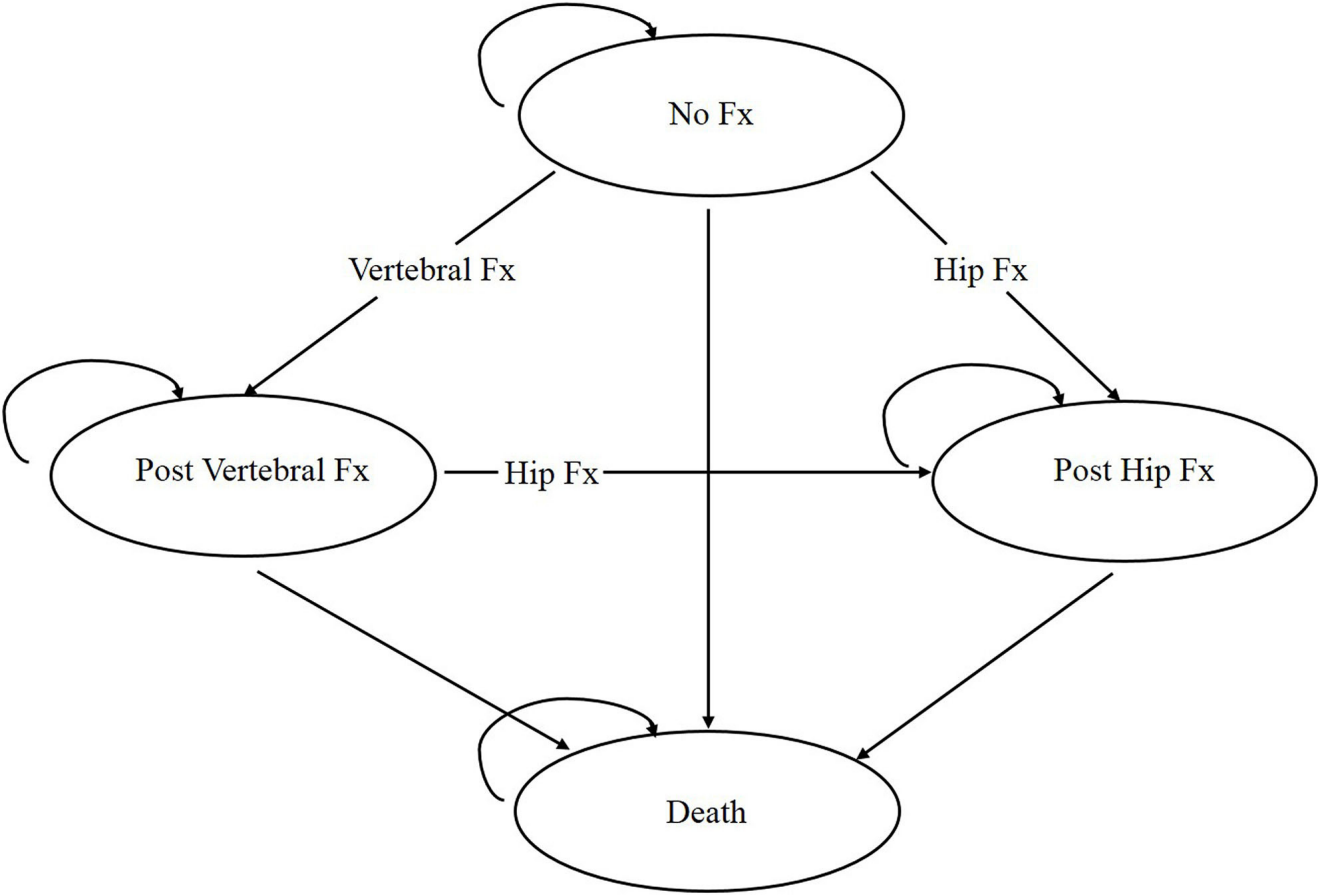
Simplified structure and transitions of the Markov model.

A Markov microsimulation model was employed to track individual characteristics and events during the simulation (e.g., number of fractures, time after the last fracture) (Figure 1). The model included four health states: no fracture, post-hip fracture, post-clinical vertebral fracture, and death. A participant starts the model in the “no fracture” state, and transitions between the health states or remains in the same state based on the assigned transition probabilities between four Markov states. A one-time cost and disutility are charged based on the participant's Markov state when a wrist or other sites of fractures happens. We set our model so that only one fracture can occur in each cycle and up to two hip fractures, but multiple other osteoporotic fractures can happen over the entire time horizon. The details of the model structure can be found in our recently published manuscripts (12, 13). Key parameters of the Markov model are presented in Table 1. For each parameter, we used data derived from peer-reviewed literature and websites that were relevant to the Chinese population, high-quality, and updated.

**Table 1:** Summary of key parameters in the model.

**Parameter**	**Value**	**Range**	**Distribution**	**References**
**Teriparatide therapy**				
Relative risk of hip fracture	0.35	0.15–0.73	Beta	(17)
Relative risk of clinical vertebral fracture	0.23	0.16–0.32	Beta	(17)
Relative risk of wrist fracture	0.24	0.02–1.00	Beta	(18)
Relative risk of other osteoporotic fracture	0.50	0.32–0.78	Beta	(18)
Adherence rate (first year)	0.70	0.60–0.80	Triangular	(19)
Persistence rate (first year)	0.68	0.58–0.78	Triangular	(19)
Treatment duration (years)	2	N/A	N/A	(20)
Offset effect (years)	2	N/A	N/A	(17)
**Zoledronic acid therapy**				
Relative risk of hip fracture	0.64	0.47–0.86	Beta	(17)
Relative risk of clinical vertebral fracture	0.40	0.29–0.55	Beta	(17)
Relative risk of wrist fracture	0.75	0.64–0.87	Beta	(12)
Relative risk of other osteoporotic fracture	0.69	0.55–0.84	Beta	(12)
Adherence rate (first year)	1.00	N/A	Triangular	(12)
Persistence rate (first year)	1.00	N/A	Triangular	(12)
Treatment duration (years)	3	N/A	N/A	(20)
Offset effect (years)	3	N/A	N/A	(17)
**Costs (2020 US dollars)**				
Annual cost for teriparatide	8,764.65	6,135.26–11,394.05	Triangular	(21, 22)
Annual cost for zoledronic acid	369.01	258.31–479.71	Triangular	(21, 22)
Hip fracture, medical costs	7,306.75	5,114.73–9,498.78	Triangular	(23)
Clinical vertebral fracture, medical costs	1,347.64	943.35–1,751.94	Triangular	(23)
Wrist fracture, medical costs	995.05	696.54–1,293.57	Triangular	(23)
Other osteoporotic fracture, medical costs	1,740.89	1,218.63–2,263.17	Triangular	(23)
Annual long-term care costs for the post-hip fracture	4,565.23	3,195.66–5,934.80	Triangular	(23)
DEXA scan	87.44	61.20–113.67	Triangular	(22)
Blood test	74.06	51.84–96.28	Triangular	(22)
Physician visit	10.29	7.20–13.37	Triangular	(22)
**Utilities**				
Age 65–69	0.806	0.765–0.846	Beta	(24)
Age 70–74	0.747	0.709–0.784	Beta	(24)
Age 75–79	0.731	0.694–0.767	Beta	(24)
Age 80–84	0.699	0.664–0.733	Beta	(24)
Age 85+	0.676	0.642–0.709	Beta	(24)
Hip fracture, first year (multiplier)	0.776	0.720–0.844	Beta	(25)
Hip fracture, subsequent year (multiplier)	0.855	0.800–0.909	Beta	(25)
Clinical vertebral fracture, first year (multiplier)	0.724	0.667–0.779	Beta	(25)
Clinical vertebral fracture, subsequent year (multiplier)	0.868	0.827–0.922	Beta	(25)
Wrist fracture (multiplier)	0.940	0.910–0.960	Beta	(26)
Other osteoporotic fracture (multiplier)	0.910	0.880–0.940	Beta	(26)
**Annual fracture incidence per 1,000 persons (without an intervention)**				
Hip fracture, age 65–69	0.96	N/A	N/A	(27)
Hip fracture, age 70–74	2.33	N/A	N/A	(27)
Hip fracture, age 75–79	4.08	N/A	N/A	(27)
Hip fracture, age 80–84	6.44	N/A	N/A	(27)
Hip fracture, age 85+	6.59	N/A	N/A	(27)
Clinical vertebral fracture, age 65–69	5.64	N/A	N/A	(28)
Clinical vertebral fracture, age 70–74	8.74	N/A	N/A	(28)
Clinical vertebral fracture, age 75–79	12.05	N/A	N/A	(28)
Clinical vertebral fracture, age 80–84	21.19	N/A	N/A	(28)
Clinical vertebral fracture, age 85+	26.89	N/A	N/A	(28)
Wrist fracture, age 65–69	12.95	N/A	N/A	(29)
Wrist fracture, age 70–74	13.17	N/A	N/A	(29)
Wrist fracture, age 75–79	13.87	N/A	N/A	(29)
Wrist fracture, age 80–84	15.01	N/A	N/A	(29)
Wrist fracture, age 85+	15.10	N/A	N/A	(29)
Other osteoporotic fracture, age 65–69	6.60	N/A	N/A	(30)
Other osteoporotic fracture, age 70–74	9.84	N/A	N/A	(30)
Other osteoporotic fracture, age 75–79	14.44	N/A	N/A	(30)
Other osteoporotic fracture, age 80–84	18.06	N/A	N/A	(30)
Other osteoporotic fracture, age 85+	26.06	N/A	N/A	(30)
**Relative risks of fractures for individuals with osteoporosis**				
Hip fracture, age 65–69	3.91	3.28–4.56	Gamma	(31, 32)
Hip fracture, age 70–74	3.13	2.80–3.47	Gamma	(31, 32)
Hip fracture, age 75–79	2.60	2.39–2.82	Gamma	(31, 32)
Hip fracture, age 80–84	2.04	1.91–2.17	Gamma	(31, 32)
Hip fracture, age 85+	1.92	1.78–2.05	Gamma	(31, 32)
Clinical vertebral fracture, age 65–69	2.59	1.19–4.27	Gamma	(32, 33)
Clinical vertebral fracture, age 70–79	2.15	1.15–3.15	Gamma	(32, 33)
Clinical vertebral fracture, age 80+	1.82	1.12–2.41	Gamma	(32, 33)
Wrist fracture, age 65–69	1.78	1.78–2.19	Gamma	(32, 33)
Wrist fracture, age 70–79	1.60	1.60–1.88	Gamma	(32, 33)
Wrist fracture, age 80+	1.45	1.45–1.64	Gamma	(32, 33)
Other osteoporotic fracture, age 65–69	2.19	1.78–2.59	Gamma	(32, 33)
Other osteoporotic fracture, age 70–79	1.88	1.60–2.15	Gamma	(32, 33)
Other osteoporotic fracture, age 80+	1.64	1.45–1.82	Gamma	(32, 33)
**Annual mortality rate**				
65–69	0.01031	N/A	N/A	(23)
70–74	0.02036	N/A	N/A	(23)
75–79	0.03784	N/A	N/A	(23)
80–84	0.06998	N/A	N/A	(23)
85+	0.13603	N/A	N/A	(23)
**Excess mortality after a hip fracture**				
Relative hazard for mortality within a year after a hip fracture	2.87	2.52–3.27	N/A	(34)
Relative hazard for mortality for second and beyond after a hip fracture	1.73	1.56–1.90	N/A	(34)
Proportion of excess mortality after a hip fracture directly attributable to a hip fracture	0.25	N/A	N/A	(35)
**Discounts**				
Costs	0.03	0–0.05	Triangular	(14)
Effectiveness	0.03	0–0.05	Triangular	(14)

### Treatment

We demonstrated the pharmacoeconomic evaluation of sequential teriparatide/zoledronic acid, which in our research was defined as daily subcutaneous teriparatide for 2 years followed by annual intravenous zoledronic acid for 3 years, compared with annual zoledronic acid monotherapy for 3 years. Due to the lack of proven efficacy beyond 2 years' use and the risk of possible osteosarcoma observed in animal studies, the use of teriparatide is approved for up to 2 years (6). Bisphosphonates are typically started after the use of teriparatide is finished in order to prevent bone density decline and loss of fracture efficacy (36). The relative risks of fractures with different therapies were based on the recently published network meta-analyses (17) and economic studies (12, 18).

In real-world practice, persistence and compliance with osteoporosis treatments are reported to be imperfect (37, 38). We considered drug persistence and compliance during the therapy based on our published papers in the Japanese or Chinese populations (12, 19). Compliance rates with teriparatide and zoledronic acid were higher in clinical than observational studies. We have constructed a microsimulation model to take into account these differences by assuming that the fracture risk reduced linearly with the increasing compliance (30, 39). After the discontinuation of the treatment, the anti-fracture benefit does not immediately disappear, but rather continues for a period of time (i.e., offset-time effect) (40). We have assumed that the offset period for teriparatide and zoledronic acid is equal to their total treatment duration and decreases proportionately. This assumption is consistent with the previous studies (30, 41). It was assumed that a individual obtained anti-fracture benefit if she insisted on medication at the end of each cycle to keep the model parsimonious. In this model, those who were not persistent with teriparatide did not start on zoledronic acid after teriparatide discontinuation.

### Fracture Incidence and Mortality Rates

The annual incidence rates of hip and clinical vertebral fractures were obtained from recent epidemiological studies in China (24). The annual incidence rates of wrist and other osteoporotic fractures were estimated using the studies in the USA and Norway as the Chinese studies were lack of the corresponding data (29, 42). We calculated the ratios of the incidences of hip fractures to those of other osteoporotic fractures using the US and Norway data and extrapolated the results to estimate the incidences of other osteoporotic fractures in China. The fracture risks were further calibrated using a method described in our prior work to improve the accuracy of the fracture risks in osteoporotic women (30, 39).

Age-specific general population mortality was informed by China Health Statistics Yearbook (23). In line with previous analyses, we assumed that hip fracture cause excess mortality (34). We conservatively assumed that only a portion (i.e., 25%) of the excess mortality following hip fracture, since we assumed comorbidities play an essential role (35). We also assumed clinical vertebral fractures scarcely contribute to excess mortality (30, 39).

### Costs

The cost of teriparatide and zoledronic acid were calculated based on the market share of generic drugs and their brand products in China from official databases of China's National Medical Products Administration (NMPA) (22) and Center for Drug Evaluation (CDE) (21). The cost of the medications were multiplied by their compliance and persistence rates with the medications. The cost of 6 months' supply of teriparatide was charged for individuals who discontinued teriparatide within 12 months. We estimated the costs for hip fracture based on previously published data in the Chinese population (23, 43). The costs of a physician visit, home healthcare, laboratory, and radiology test were obtained from the National Development and Reform Commission of China (44). Costs from past sources were converted to 2020 US dollars ($1 = 6.8974 Chinese yuan [¥]) and were inflated to 2020 price using the Consumer Price Index (CPI) of China (45).

### Utilities

We estimated health-related quality of life (HRQoL) of individuals in the baseline by age-specific EQ-5D scores from the Chinese National Health Services Survey (24). To account for the HRQoL loss with a fracture, utility multipliers were applied in the first year following all types of fractures and the second and subsequent years following hip and vertebral fractures (26, 30).

### Model Simulation and Sensitivity

We conducted the base case including NMB and NHB, and one-way deterministic and probabilistic sensitivity analyses (PSA). One-way sensitivity analysis was conducted to examine how alternative parameters influence on the ICERs. PSA was conducted to assess the joint uncertainty across the ranges of virtually all the essential parameters by performing Monte-Carlo simulations (1,000 simulations and 10,000 trials per simulation). In the PSA, probability distributions for each parameter were assigned rather than point estimates to assess the uncertainty of the parameters.

## Results

### Model Validation

Without an intervention, our model estimated that the probabilities of dying by age 105 were >99% in each starting age (i.e., 65, 70, 75, and 80), in keeping with those in the 2020 Chinese life table (46). In addition, our research predicted that without an intervention, the cumulative probabilities of having hip or clinical vertebral fracture at least once were 11.50 or 39.69%, respectively, consistent with China's epidemiological data (20).

### Base Case Analysis

The total costs, number of fractures, QALYs, and ICERs, calculated in the model at different initial ages were presented in Table 2. As an example of age 65 years, compared with no treatment (mean cost $5,933.70; mean effect 9.36 QALYs), zoledronic acid monotherapy was shown to be cost-saving with less costs, more health benefits, and more fractures prevented. However, sequential teriparatide/zoledronic acid (mean cost $10,039.29; mean effect 9.52 QALYs) was associated with higher health care cost of $5,196.69 and QALY of 0.03 compared with zoledronic acid monotherapy, yielded in an ICER of $173,223.00/QALY gained. Moreover, both NMB and NHB were negative, which further showed that zoledronic acid monotherapy was more cost-effective than sequential teriparatide/zoledronic acid at the WTP threshold of $31,512/QALY. The results at other initial ages (i.e., 70, 75, or 80) were similar to those, and the conclusions remained the same.

**Table 2:** Base case results at various ages of therapy initiation.

	**No treatment**	**TPTD/ZOL**	**ZOL MONO**	**TPTD/ZOL vs. ZOL MONO**
**Aged 65 years**				
Total costs (2020 US Dollars)	5,933.70	10,039.29	4,842.60	5,196.69
Healthcare costs	5,933.70	3,647.56	3,727.59	−80.03
Treatment costs	0	6,391.73	1,115.01	5,276.72
QALYs	9.36	9.52	9.49	0.03
Number of all fractures	1.9794	1.4969	1.5513	−0.0544
ICER ($/QALY gained)				173,223.00
NMB				−4,251.33
NHB				−0.13
**Aged 70 years**				
Total costs (2020 US Dollars)	5,482.34	10,001.46	4,737.23	5,264.23
Healthcare costs	5,482.34	3,349.23	3,629.25	−280.02
Treatment costs	0	6,652.23	1,107.98	5,544.25
QALYs	7.51	7.74	7.70	0.04
Number of all fractures	1.7780	1.3760	1.4140	−0.0380
ICER ($/QALY gained)				131,605.75
NMB				−4,003.75
NHB				−0.12
**Aged 75 years**				
Total costs (2020 US Dollars)	4,833.88	9,012.55	4,018.52	4,994.03
Healthcare costs	4,833.88	2,801.26	2,932.08	−130.82
Treatment costs	0	6,211.29	1,086.44	5,124.85
QALYs	5.81	5.99	5.94	0.05
Number of all fractures	1.5514	1.0680	1.1180	−0.0500
ICER ($/QALY gained)				99,880.60
NMB				−3,418.43
NHB				−0.11
**Aged 80 years**				
Total costs (2020 US Dollars)	4,401.93	8,794.69	3,655.48	5,139.21
Healthcare costs	4,401.93	2,621.44	2,612.82	8.62
Treatment costs	0	6,173.25	1,042.66	5,130.59
QALYs	4.40	4.68	4.59	0.09
Number of all fractures	1.3010	0.9560	1.0140	−0.0580
ICER ($/QALY gained)				57,102.33
NMB				−2,303.13
NHB				−0.07

*TPTD/ZOL, sequential teriparatide/zoledronic acid; ZOL MONO, zoledronic acid monotherapy; US Dollars, United States Dollars; QALYs, quality-adjusted life years; ICER, incremental cost-effectiveness ratio; NMB, net monetary benefit; NHB, net health benefit*.

### One-Way Sensitivity Analysis

In one-way sensitivity analyses, the two most impactful parameters were the residual effects for the treatments and the cost of teriparatide at the age of 65 (Table 3). Assuming without the residual effects, the ICER would increase to $257,982.00/QALY. It is worth noting that the drug costs of teriparatide reduced by 50% for individuals aged 80 changed the base case results. ICER was markedly decreased to $70,571.33/QALY when the drug costs of teriparatide were reduced by 50% (Supplementary Table 1).

**Table 3:** Results of one-way sensitivity analyses at 65 years.

**Parameter**	**Cost (2020 US Dollars)**		**ΔC**	**Effectiveness (QALYs)**		**ΔE**	**ICER ($/QALY gained)**
	**TPTD/ZOL**	**ZOL MONO**		**TPTD/ZOL**	**ZOL MONO**		
No residual effect	10,131.94	4,972.30	5,159.64	9.44	9.42	0.02	257,982.00
10-year time horizon	9,781.11	4,773.89	5,007.22	9.45	9.41	0.04	125,180.50
TPTD persistence rate 10% higher	11,553.33	4,752.95	6,800.38	9.41	9.36	0.05	136,007.60
Discount rate 0%	10,560.52	5,221.91	5,338.61	12.29	12.26	0.03	177,953.67
Discount rate 5%	9,716.89	4,805.15	4,911.74	8.15	8.12	0.03	163,724.67
Fracture costs 30% higher	10,199.95	4,908.31	5,291.64	9.52	9.49	0.03	176,388.00
Fracture costs 30% lower	9,804.78	4,763.04	5,041.74	9.47	9.44	0.03	168,058.00
TPTD cost 30% lower	8,306.46	4,751.16	3,555.30	9.50	9.48	0.02	177,765.00
TPTD cost 50% lower	7,158.67	5,041.53	2,117.14	9.32	9.29	0.03	70,571.33
Excess mortality 50% higher	9,790.73	4,616.91	5,173.82	9.37	9.33	0.04	129,345.50
Excess mortality 0%	10,276.41	4,852.31	5,424.10	9.41	9.38	0.03	180,803.33

*TPTD/ZOL, sequential teriparatide/zoledronic acid; ZOL MONO, zoledronic acid monotherapy; US Dollars, United States Dollars; QALYs, quality-adjusted life years; ICER, incremental cost-effectiveness ratio*.

### Probabilistic Sensitivity Analysis

**Figure 2:**
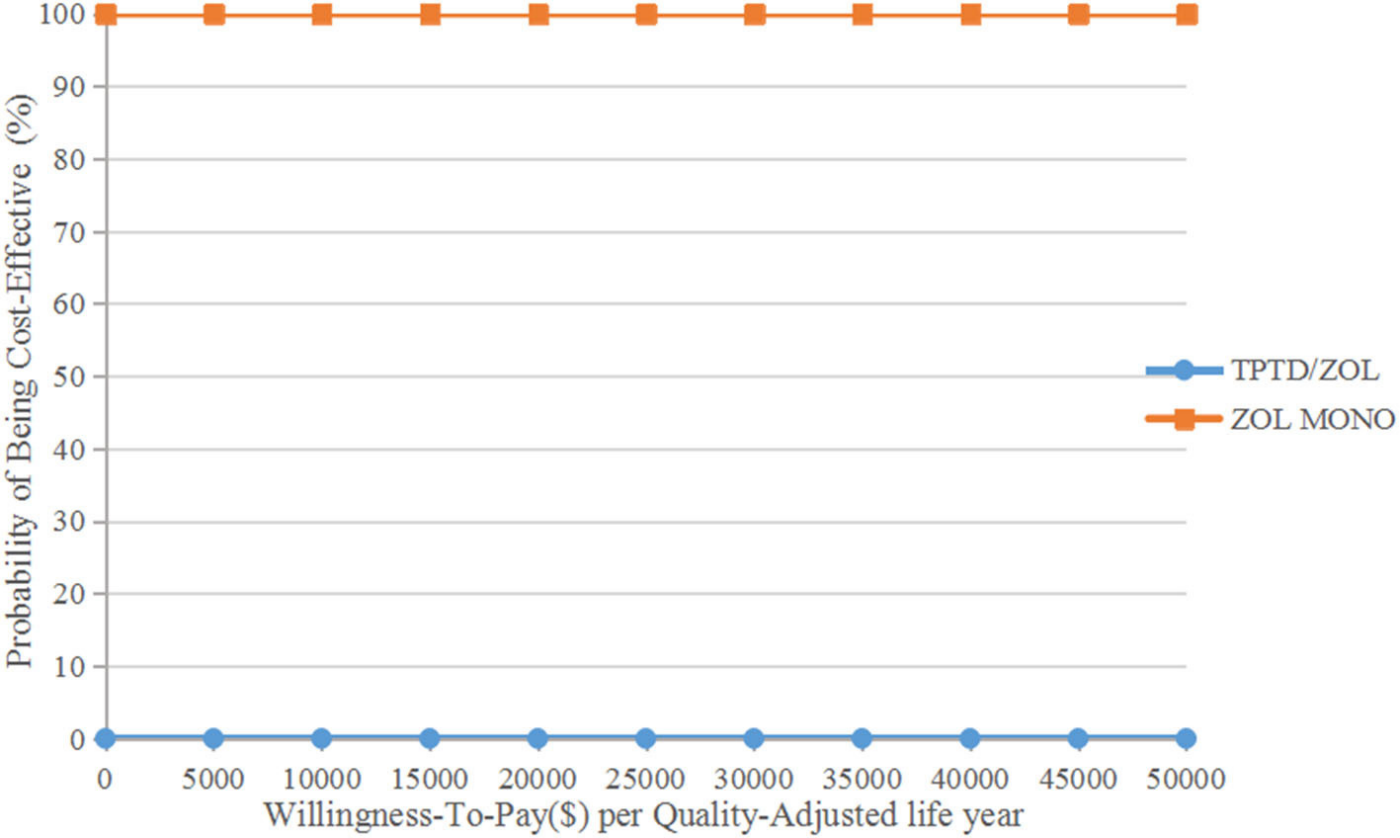
Results of probabilistic sensitivity analyses age 65 years. The cost-effectiveness acceptability curves represent probabilities of being cost-effective achieved by the sequential teriparatide/zoledronic acid strategy relative to the zoledronic acid monotherapy strategy.

The aforementioned results were further validated in probabilistic sensitivity analyses. At a threshold of $31,512/QALY, the probabilities of zoledronic acid monotherapy being cost-effective were 100% for ages 65 and 70 and 98% for ages 75 and 80, respectively (Figure 2; Supplementary Figures 1–3).

## Discussions

Sequential therapy beginning with teriparatide followed by zoledronic acid was not a cost-effective strategy relative to annual intravenous zoledronic acid monotherapy for postmenopausal osteoporotic women. One-way and probabilistic sensitivity analyses indicated that the results were robust. It is worth noting that the drug costs of teriparatide reduced by 50% for individuals aged 80 would lead to different results from the base case. In this situation, the ICER of sequential teriparatide/zoledronic acid relative to zoledronic acid monotherapy declined to $29,476/QALY and was below the pre-determined threshold. As in a previous study in the US, we found that one of the key parameters of sequential teriparatide/alendronate not being cost-effective was the high cost of teriparatide. Hence, we examined how further discounts on the costs of teriparatide would affect cost-effectiveness.

Evidence accumulated over the past decade supports that sequential therapy with the initiation of anabolic treatment followed by antiresorptive treatment improves health-related quality-of-life outcomes for individuals with osteoporosis (47) and our current study reinforces this strategy from the health economic points of view. In the earlier published work, one of the authors of the current study compared sequential daily teriparatide/weekly alendronate with alendronate monotherapy in older osteoporotic women with a prior vertebral fracture in the US (18) and Japan (19). Both studies demonstrated that even with the availability of generic teriparatide, sequential teriparatide/alendronate would not be cost-effective unless the cost of generic teriparatide was largely discounted compared with the brand one. Our current study confirms and extends prior work in this area in the China setting. First, unlike previous studies, we choose zoledronic acid after the completion of teriparatide. Intravenous zoledronic acid has become a better replacement for oral alendronate due to the higher persistence and compliance. In addition, in our model, we added wrist and other osteoporotic fractures, which are the common fractures among the target population. If a participant had a wrist or other bone fracture, corresponding one-time cost and disutility are assigned.

Some more pharmacoeconomic evaluations regarding sequential therapy have been reported, which were based on the US or Japanese settings (40, 48, 49). These two studies in the United States demonstrated that abaloparatide followed by alendronate was dominant compared with sequential teriparatide/alendronate and was cost-effective compared with alendronate monotherapy. As abaloparatide was not available for osteoporosis in China at the time of the current study, our model did not include it. As we focused on sequential treatment beginning with the anabolic agent followed by the antiresorptive agent in this analysis, we did not assess cost-effectiveness of sequential treatment with the beginning of an antiresorptive agent followed by another antiresorptive agent.

There are still certain limitations in our current economic analysis. Firstly, our results and conclusion should be generalized conservatively to healthcare settings other than China. The major issue regarding generalizability is whether costs and effectiveness of interventions vary across different settings or populations. In addition, although most of the parameter inputs in this model were based on the Chinese population, some inputs were extrapolated from other populations. An updated cost-effectiveness analysis should be conducted when these parameter inputs are available in the Chinese context. Second, although in the base case we assumed that teriparatide was effective to reduce the risk of hip fracture, it should be noted that the literature on teriparatide regarding the prevention of hip fracture is limited. In the real-world setting, the fracture risk reduction of the medication compared with placebo may be different from that in the clinical trials. In addition, the comparison between sequential teriparatide/zoledronic acid and zoledronic acid monotherapy should be interpreted with caution as these two sequential strategies were not compared head-to-head in the same clinical trial. However, modeling offers the advantage and is commonly used to assess the cost-effectiveness of interventions that have not been compared directly in the same trial. Third, to keep our model not too complicated, we did not consider the issue of adverse events and some contraindications. However, serious adverse events caused by current strategies for the treatment of osteoporosis are considered to be rare (20); therefore, we do not believe they have a meaningful impact on the conclusion of our current study. Furthermore, although the offset periods of the mediations may be different, in order to make the model concise and easy to operate we have assumed that the offset periods for each medication are the same, which is also one of the limitations.

Despite of these limitations, this research also has some advantages. First, to our knowledge, this is the first substantial study to evaluate sequential therapy in osteoporosis based on a Chinese setting. In addition, we confirmed that the key driving factor of sequential teriparatide/zoledronic acid not being cost-effective was the high price of teriparatide. Second, we included compliance and persistence with the treatments into our economic analysis and investigated how they impact on our model results, as poor compliance and persistence have been reported to be essential parameters in pharmacoeconomic analyses for current osteoporosis therapies.

In conclusion, from the perspective of Chinese health care payer, annual intravenous zoledronic acid monotherapy for 3 years was cost-effective compared with sequential daily subcutaneous teriparatide for 2 years followed by annual intravenous zoledronic acid for 3 years in Chinese women with postmenopausal osteoporosis at the pre-determined WTP threshold of $31,512/QALY. This study provides practical and useful points of view for clinicians as well as policymakers regarding osteoporosis treatment in older Chinese women.

## Data Availability Statement

The original contributions presented in the study are included in the article/Supplementary Material, further inquiries can be directed to the corresponding author/s.

## Ethics Statement

The studies involving human participants were reviewed and approved by the Institutional Ethics Committee of Tongji Medical College of Huazhong University of Science and Technology, Wuhan, China. As this economic analysis was based on a literature review and modeling techniques, it was exempted from consent procedure by the Institutional Ethics Committee of Tongji Medical College of Tongji Medical College of Huazhong University of Science and Technology. Written informed consent for participation was not required for this study in accordance with the national legislation and the institutional requirements.

## Author Contributions

RY and TM: study design and revising manuscript content. RY and JL: study conduct and drafting manuscript. LK and GY: data collection. RY, JL, and TM: data analysis and approving final version of manuscript. RY, JL, LK, GY, YZ, and TM: data interpretation. RY: takes responsibility for the integrity of the data analysis. All authors contributed to the article and approved the submitted version.

## Conflict of Interest

The authors declare that the research was conducted in the absence of any commercial or financial relationships that could be construed as a potential conflict of interest.

## Publisher's Note

All claims expressed in this article are solely those of the authors and do not necessarily represent those of their affiliated organizations, or those of the publisher, the editors and the reviewers. Any product that may be evaluated in this article, or claim that may be made by its manufacturer, is not guaranteed or endorsed by the publisher.

## Supplementary Material

Supplementary Figure 1 Results of probabilistic sensitivity analyses, age 70 years.

Supplementary Figure 2 Results of probabilistic sensitivity analyses, age 75 years.

Supplementary Figure 3 Results of probabilistic sensitivity analyses, age 80 years.

Supplementary Table 1 Results of one-way analyses of at 65, 70, and 80 years.
