# Supplementary figures and images for: Cost-Effectiveness of Sequential Teriparatide/Zoledronic Acid Compared With Zoledronic Acid Monotherapy for Postmenopausal Osteoporotic Women in China
Source: Front Public Health. 2022 Feb 24;10:794861. doi: 10.3389/fpubh.2022.794861 (PMC8907523; doi:10.3389/fpubh.2022.794861)

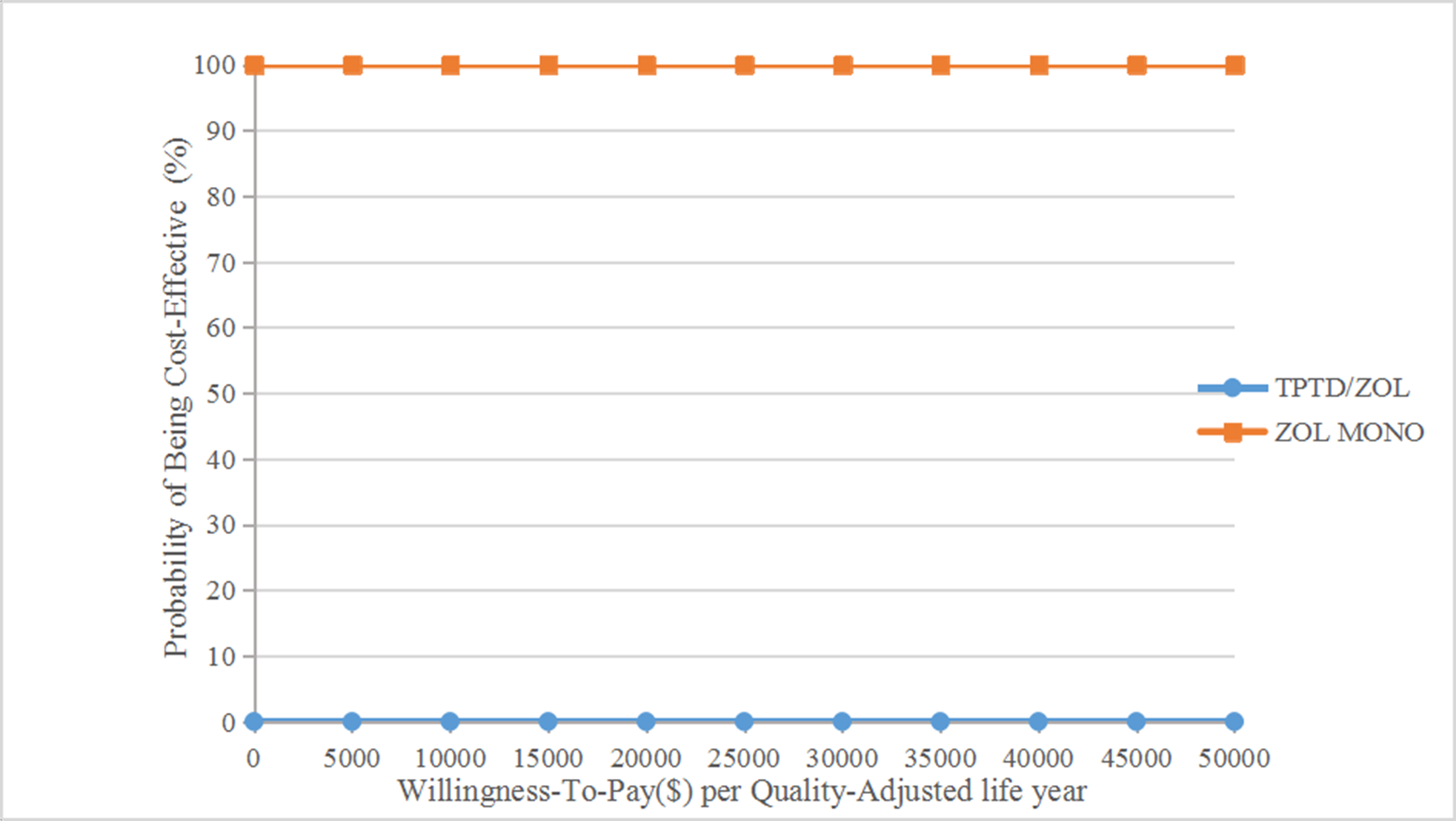

Supplement: Supplementary Figure 1 — Results of probabilistic sensitivity analyses, age 70 years. [file Image_1.tif]

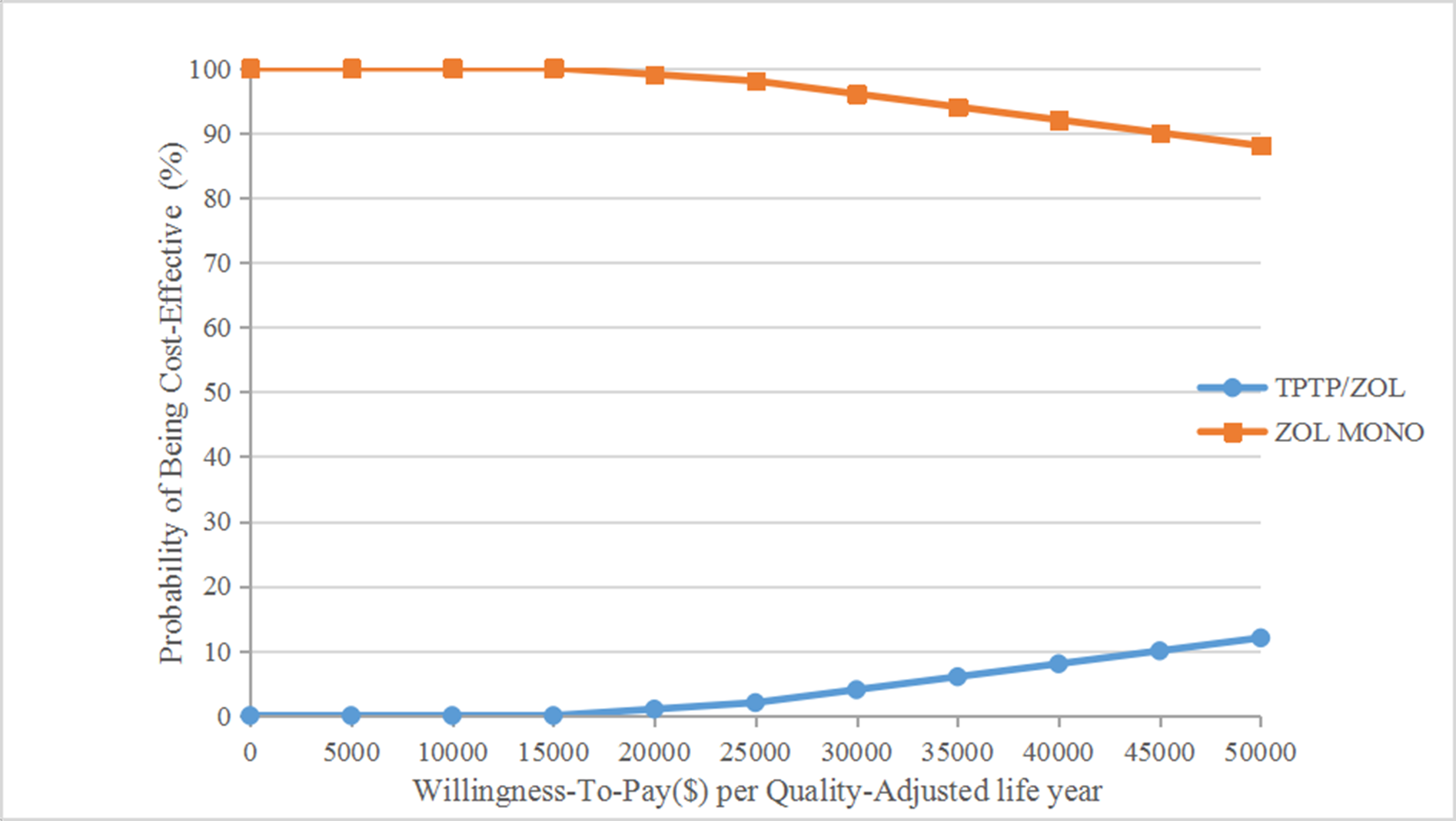

Supplement: Supplementary Figure 2 — Results of probabilistic sensitivity analyses, age 75 years. [file Image_2.tif]

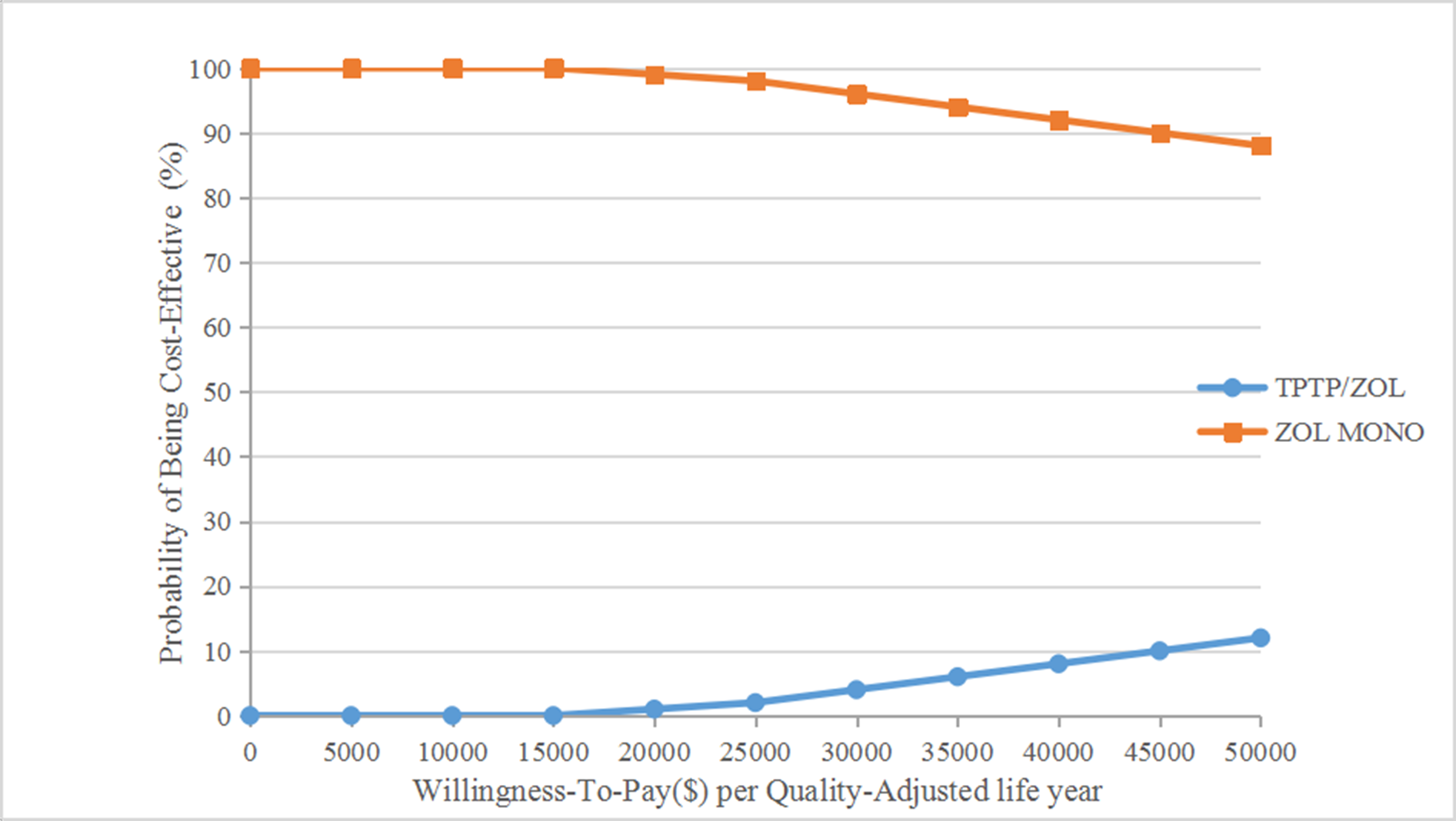

Supplement: Supplementary Figure 3 — Results of probabilistic sensitivity analyses, age 80 years. [file Image_3.tif]
