# Supplementary material for: Cost-Effectiveness of Sequential Teriparatide/Zoledronic Acid Compared With Zoledronic Acid Monotherapy for Postmenopausal Osteoporotic Women in China
Source: Front Public Health. 2022 Feb 24;10:794861. doi: 10.3389/fpubh.2022.794861 (PMC8907523; doi:10.3389/fpubh.2022.794861)
Supplement: Supplementary Table 1 — Results of one-way analyses of at 65, 70, and 80 years. [file Table_1.DOCX]

Supplemental Table 1 Results of one-way analyses of at 65, 70, and 80 years.

| **Parameter** | **Cost (2020 US Dollars)** | | **△C** | **Effectiveness (QALYs)** | | **△E** | **ICER**  **($/QALY gained)** |
| --- | --- | --- | --- | --- | --- | --- | --- |
|  | TPTD/ZOL | ZOL MONO |  | TPTD/ZOL | ZOL MONO |  |  |
| **Aged 70 years** |  |  |  |  |  |  |  |
| No residual effect | 9772.64 | 4537.51 | 5235.13 | 7.61 | 7.58 | 0.03 | 174504.33 |
| 10-year time horizon | 9739.62 | 4303.07 | 5436.55 | 7.56 | 7.51 | 0.05 | 108731.00 |
| TPTD persistence rate 10% higher | 11322.95 | 4383.11 | 6939.84 | 7.62 | 7.57 | 0.05 | 138796.80 |
| Discount rate 0% | 10656.91 | 4868.87 | 5788.04 | 9.38 | 9.33 | 0.05 | 115760.80 |
| Discount rate 5% | 9428.08 | 4138.49 | 5289.59 | 6.64 | 6.60 | 0.04 | 132239.75 |
| Post-hip fracture costs 30% higher | 9688.23 | 4279.62 | 5408.61 | 7.47 | 7.43 | 0.04 | 135215.25 |
| Post-hip fracture costs 30% lower | 9246.94 | 4218.68 | 5028.26 | 7.52 | 7.48 | 0.04 | 125706.50 |
| TPTD costs 30% lower | 7906.66 | 4437.42 | 3469.24 | 7.70 | 7.66 | 0.04 | 86731.00 |
| TPTD costs 50% lower | 6761.47 | 4311.12 | 2450.35 | 7.62 | 7.58 | 0.04 | 61258.75 |
| Excess mortality 50% higher | 9584.98 | 4537.73 | 5047.25 | 7.52 | 7.47 | 0.05 | 100945.00 |
| Excess mortality 0% | 9920.63 | 4229.17 | 5691.46 | 7.47 | 7.43 | 0.04 | 142286.50 |
| **Aged 75 years** |  |  |  |  |  |  |  |
| No residual effect | 9358.64 | 4176.08 | 5182.56 | 5.90 | 5.86 | 0.04 | 129564.00 |
| 10-year time horizon | 8701.85 | 3889.44 | 4812.41 | 5.83 | 5.78 | 0.05 | 96248.20 |
| TPTD persistence rate 10% higher | 10766.37 | 4142.53 | 6623.84 | 5.98 | 5.91 | 0.07 | 94626.29 |
| Discount rate 0% | 9424.66 | 4206.66 | 5218.00 | 7.45 | 7.40 | 0.05 | 104360.00 |
| Discount rate 5% | 8856.66 | 3828.54 | 5028.12 | 5.43 | 5.39 | 0.04 | 125703.00 |
| Post-hip fracture costs 30% higher | 8826.88 | 4306.79 | 4520.09 | 6.13 | 6.09 | 0.04 | 113002.25 |
| Post-hip fracture costs 30% lower | 8762.17 | 3966.60 | 4795.57 | 6.00 | 5.95 | 0.05 | 95911.40 |
| TPTD costs 30% lower | 7494.14 | 3737.28 | 3756.86 | 5.84 | 5.79 | 0.05 | 75137.20 |
| TPTD costs 50% lower | 6198.35 | 3968.44 | 2229.91 | 5.88 | 5.83 | 0.05 | 44598.20 |
| Excess mortality 50% higher | 9306.95 | 4115.90 | 5191.05 | 6.02 | 5.97 | 0.05 | 103821.00 |
| Excess mortality 0% | 9404.17 | 4020.45 | 5383.72 | 6.08 | 6.04 | 0.04 | 134593.00 |
| **Aged 80 years** |  |  |  |  |  |  |  |
| No residual effect | 8064.00 | 3512.45 | 4551.55 | 4.50 | 4.46 | 0.04 | 113788.75 |
| 10-year time horizon | 8297.25 | 3601.89 | 4695.36 | 4.53 | 4.44 | 0.09 | 52170.67 |
| TPTD persistence rate 10% higher | 10328.65 | 3521.53 | 6807.12 | 4.73 | 4.61 | 0.12 | 56726.00 |
| Discount rate 0% | 8887.76 | 3694.59 | 5193.17 | 4.79 | 4.69 | 0.10 | 51931.70 |
| Discount rate 5% | 8555.75 | 3503.96 | 5051.79 | 4.52 | 4.43 | 0.09 | 56131.00 |
| Post-hip fracture costs 30% higher | 8651.42 | 3735.54 | 4915.88 | 4.68 | 4.62 | 0.06 | 81931.33 |
| Post-hip fracture costs 30% lower | 8104.71 | 3539.78 | 4564.93 | 4.43 | 4.36 | 0.07 | 65213.29 |
| TPTD costs 30% lower | 6849.50 | 3363.59 | 3485.91 | 4.63 | 4.56 | 0.07 | 49798.71 |
| TPTD costs 50% lower | 5875.87 | 3517.82 | 2358.05 | 4.62 | 4.54 | 0.08 | 29475.63 |
| Excess mortality 50% higher | 8427.96 | 3401.44 | 5026.52 | 4.45 | 4.35 | 0.10 | 50265.20 |
| Excess mortality 0% | 8927.51 | 3593.41 | 5334.10 | 4.57 | 4.49 | 0.08 | 66676.25 |

Abbreviations: TPTD/ZOL, sequential teriparatide/zoledronic acid; ZOL MONO, zoledronic acid monotherapy; US Dollars, United States Dollars; QALYs, quality-adjusted life years; ICER, incremental cost-effectiveness ratio
